# Germline *POLE* and *POLD1* proofreading domain mutations in endometrial carcinoma from Middle Eastern region

**DOI:** 10.1186/s12935-019-1058-9

**Published:** 2019-12-11

**Authors:** Abdul K. Siraj, Sandeep Kumar Parvathareddy, Rong Bu, Kaleem Iqbal, Sarah Siraj, Tariq Masoodi, Rica Micaela Concepcion, Laila Omar Ghazwani, Ismail AlBadawi, Fouad Al-Dayel, Khawla S. Al-Kuraya

**Affiliations:** 1 Human Cancer Genomic Research, Research Center, King Faisal Specialist Hospital and Research Center, MBC#98-16, P.O. Box 3354, Riyadh, 11211 Saudi Arabia; 2 Department of Obstetrics-Gynecology, King Faisal Specialist Hospital and Research Center, Riyadh, Saudi Arabia; 3 Department of Pathology, King Faisal Specialist Hospital and Research Centre, P.O. Box 3354, Riyadh, 11211 Saudi Arabia

**Keywords:** Endometrial carcinoma, *POLE*, *POLD1*, Proofreading domains

## Abstract

**Background:**

Endometrial carcinoma (EC) accounts for 5.8% of all cancers in Saudi females. Although most ECs are sporadic, 2–5% tend to be familial, being associated with Lynch syndrome and Cowden syndrome. In this study, we attempted to uncover the frequency, spectrum and phenotype of germline mutations in the proofreading domain of *POLE* and *POLD1* genes in a large cohort of ECs from Middle Eastern region.

**Methods:**

We performed Capture sequencing and Sanger sequencing to screen for proofreading domains of *POLE* and *POLD1* genes in 432 EC cases, followed by evaluation of protein expression using immunohistochemistry. Variant interpretation was performed using PolyPhen-2, MutationAssessor, SIFT, CADD and Mutation Taster.

**Results:**

In our cohort, four mutations (0.93%) were identified in 432 EC cases, two each in *POLE* and *POLD1* proofreading domains. Furthermore, low expression of POLE and POLD1 was noted in 41.1% (170/1414) and 59.9% (251/419) of cases, respectively. Both the cases harboring *POLE* mutation showed high nuclear expression of POLE protein, whereas, of the two *POLD1* mutant cases, one case showed high expression and another case showed low expression of POLD1 protein.

**Conclusions:**

Our study shows that germline mutations in *POLE* and *POLD1* proofreading region are a rare cause of EC in Middle Eastern population. However, it is still feasible to screen multiple cancer related genes in EC patients from Middle Eastern region using multigene panels including *POLE* and *POLD1*.

## Background

Endometrial carcinoma (EC) is the second most common gynecologic cancer Worldwide, with its annual incidence projected to increase [1]. In Saudi Arabia, EC is the fourth most common malignancy among women, accounting for 5.8% of all cancers in females [2]. Numerous genetic mutations have been discovered in the past few years leading to a better understanding of hereditary syndromes associated with malignancies of the female genital tract [3]. Although majority of ECs are sporadic, 2–5% tend to be familial [4]. Familial EC has been linked to germline mutations in the mismatch repair genes associated with Lynch Syndrome (LS), or to germline mutations in *PTEN* associated with Cowden Syndrome [5, 6]. A recent study has shown that germline missense mutations of *POLE* and *POLD1* genes lead to development of polymerase proofreading-associated polyposis, which is similar to LS with regards to tumor spectrum, including an increased risk of ECs [7].

*POLE* and *POLD1* are related B family polymerases. They form the major catalytic and proofreading subunits of the DNA polymerase Epsilon (*Polε*) and DNA polymerase Delta (*Polδ*) enzyme complexes [8]. Both *Polε* and *Polδ* are heterotetramers with Polymerase ε involved in replication of leading strand of the replication fork [9], whereas DNA polymerase δ functions in synthesizing the lagging strand [10]. Both polymerases δ and ε are responsible for carrying out high fidelity DNA synthesis and mutation affecting the proofreading activity of these genes can lead to genome instability, and subsequent increased risk of developing cancer [11].

*POLE* mutations constitute a specific molecular subgroup of EC, and have both prognostic and therapeutic implications for the patient [12]. The Cancer Genome Atlas (TCGA) characterized 373 cases of EC, based on their integrated genomic, transcriptomic, and proteomic data, into four molecular subgroups. Tumors with *POLE* mutations were identified as one of the subgroups and represented an ultra-mutated tumor phenotype [12]. Somatic mutations of *POLE* gene have been reported in 6–10% of ECs and 1–2% of colorectal cancers [12–15]. Few cases of lung, breast, stomach, pancreatic, brain and ovarian tumors have also been shown to harbor these mutations [16, 17]. Although rare, germline *POLE* mutations have been reported in 0.25–4% of ECs [18–20].

Currently, there is no known prognostic significance associated with *POLD1* mutation. Instead, emphasis is placed on identification of *POLD1* germline mutations due to the potential risk of developing secondary tumors in a hereditary syndromic manner [21].

With the advent of individualized therapy, greater emphasis is placed on identifying specific genetic alterations and molecular subtypes of EC. In this study, we report the frequency, spectrum and phenotype of germline mutations in the proofreading domains of *POLE* and *POLD1* genes in a large cohort of ECs from Middle Eastern region. It may contribute to a better understanding of the molecular mechanisms underlying EC and could also have important preventive and/or therapeutic implications in Middle Eastern population.

## Materials and methods

### Sample selection

Archival samples from 432 EC patients diagnosed between 1990 and 2016 at King Faisal Specialist Hospital and Research Center (Riyadh, Saudi Arabia) were included in the study. Detailed clinicopathological data were noted from case records and have been summarized in Table 1. All samples were obtained from patients with approval from Institutional Review Board of the hospital. For the study, waiver of consent was obtained for archived paraffin tissue blocks from Research Advisory Council (RAC) under project RAC# 2180 001.

**Table 1:** Clinicopathological variables for the patient cohort (n = 432)

Clinico-pathological parameter	n (%)
Age	
Median	59.3
Range (IQR)	53.0–66.0
Histologic subtype	
Type I	370 (88.1)
Type II	50 (11.9)
Histological grade	
Well differentiated	146 (33.8)
Moderately differentiated	145 (33.6)
Poorly differentiated	128 (29.6)
Unknown	13 (3.0)
TNM stage	
I	278 (64.3)
II	47 (10.9)
III	69 (16.0)
IV	37 (8.6)
Unknown	1 (0.2)

*IQR* inter quartile range

### DNA extraction

DNAs were isolated from formalin-fixed, paraffin-embedded (FFPE) EC non-tumor tissues using Gentra DNA isolation kit (Gentra, Minneapolis, MN, USA) following the manufacturer’s recommendations as described previously [22].

### Targeted capture sequencing of germline mutations in proofreading domain of POLE and POLD1 genes

The capture sequencing was performed on 53 EC cases as described previously [23]. The DNA samples with A260/A280 ratio between 1.8 and 2.0 were processed for library construction. The sequencing library was prepared by random fragmentation of the DNA, followed by 5′ and 3′ adapter ligation. Adapter-ligated fragments were then PCR amplified and gel purified. Clusters were generated by loading the library into a flow cell where fragments were captured on a lawn of surface-bound oligos complementary to the library adapters. Each fragment was then amplified into distinct, clonal clusters through bridge amplification. Raw data was generated utilizing HCS (HiSeq control software v3.3) and RTA (real-time analysis. v2.5.2).

The BCL (base calls) generated by Illumina Hiseq 4000 were converted into FASTQ files by bcl2fastq (v2.16). The sequence reads in fastq format from each sample were aligned to the reference human genome (GRCh37/hg19) using burrows-wheeler aligner (BWA) [24]. BAM file generation, PCR duplicates and local realignment was performed using Picard-tools and genome analysis toolkit (GATK) [25].

The variant calling was performed by GATK, subsequently the variants were annotated by ANNOVAR [26], with dbSNP138, 1000 Genomes, ESP6500, Exome Aggregation Consortium (ExAC), Clinvar and other genome databases.

### Polymerase Chain Reaction (PCR) and Sanger Sequencing for detection of Germline Mutations in Proofreading Domain of POLE and POLD1 genes

Direct sequencing of the entire coding/splicing region of proofreading domain of *POLE* and *POLD1* genes were performed on 379 samples. In addition, detected mutations by Capture sequencing were further confirmed by Sanger sequencing in 53 cases. Primer 3 software was used to design the primers for all coding exons and their flanking intronic sequences of proofreading domain of *POLE* and *POLD1* genes (available upon request). PCR was performed in a total volume of 25 μL using 20 ng of genomic DNA, 2.5 μL 10× Taq buffer, 2.3 mM MgCl_2_, 0.2 mM dNTPs, 1 unit Taq polymerase (all reagents were from Qiagen Inc), 0.2 μM of each primer, and water. The efficiency and quality of the amplified PCR products were confirmed by running the PCR products on a 2% agarose gel.

For Sanger sequencing, the PCR products were subsequently subjected to direct sequencing with BigDye terminator V 3.1 cycle sequencing reagents and analyzed on an ABI 3730XL DNA analyzer (Applied Biosystems, Foster City, CA). Reference sequences were downloaded from NCBI GenBank. Sequencing results were compared with the reference sequences by Mutation Surveyor V4.04 (Soft Genetics, LLC, State College, PA).

### Assessment of Pathogenicity of Variants

ACMG/AMP 2015 guideline was utilized first for interpretation of sequence variants [27]. All the uncertain significant variants interpreted by ACMG/AMP 2015 guideline were further analyzed using five in silico pathogenicity prediction tools: PolyPhen-2 [28], MutationAssessor [29], SIFT [30], CADD [31] and Mutation Taster [32]. The variants predicted as damaging or possibly damaging by three or more in silico prediction tools were considered as pathogenic mutations.

### Tissue microarray construction and Immunohistochemistry

All samples were analyzed in a tissue microarray (TMA) format. TMA construction was performed as described earlier [33]. Briefly, tissue cylinders with a diameter of 0.6 mm were punched from representative tumor regions of each donor tissue block and brought into recipient paraffin block using a modified semiautomatic robotic precision instrument (Beecher Instruments, Woodland, WI). Two cores of EC were arrayed from each case.

Standard protocol was followed for immunohistochemistry (IHC) staining. For antigen retrieval, Dako (Dako Denmark A/S, Glostrup, Denmark) Target Retrieval Solution pH 9.0 (Catalog number S2367) was used, and the slides were placed in Pascal pressure cooker for 8 min at 120 °C. The slides were incubated with primary antibodies against POLE (ab-134941, Abcam, Cambridge, UK) and POLD1 (ab-186407, Abcam, Cambridge, UK) at a dilution of 1:1000 (pH 9.0). The Dako Envision Plus System kit was used as the secondary detection system with 3, 30-diaminobenzidine as chromogen. All slides were counterstained with hematoxylin, dehydrated, cleared and mounted. Negative controls included omission of the primary antibody. Normal tissues of different organ systems were also included in the TMA to serve as control. Only fresh cut slides were stained simultaneously to minimize the influence of slide aging and maximize reproducibility of the experiment.

Each TMA spot was assigned an intensity score from 0 to 3 (I0–I3) corresponding to no, weak, moderate and strong staining, and the proportion of tumor staining for that intensity was recorded as 5% increments from a range of 0–100 (P0–P3). A final H score (range 0–300) was obtained by adding the products of scores obtained for each intensity and proportion of area stained (H score = I1XP1 + I2XP2 + I3XP3). Using X-tile version 3.6.1 [34], we defined the optimal cutoff point for POLE and POLD1 expression as H = 90 and H = 175, respectively. Based on H scores, EC cases were classified into two subgroups: those below the cutoff score were defined as low expression and those above the cutoff score were defined as over expression.

Staining and evaluation of mismatch repair proteins (MLH1, MSH2, MSH6 and PMS2) was performed as described previously [35].

### Statistical analysis

Contingency table analysis and Chi square tests were used to study the relationship between clinico-pathological variables and protein expression or mutation. Overall Survival curves were generated using the Kaplan–Meier method, with significance evaluated using the Mantel–Cox log-rank test. The limit of significance for all analyses was defined as p value of < 0.05; two-sided tests were used in these calculations. The JMP11.0 (SAS Institute, Inc., Cary, NC) software package was used for data analyses.

## Results

### Sample characteristics

A total of 432 EC cases were analyzed. Median age of the study cohort was 59 years. Tumors were predominantly of type I EC (88.1%) with an almost equal distribution among the three grades. Majority of the cases were Stage I tumors (64.3%) (Table 1).

### Identification of Germline Mutations in Proofreading Domain of POLE and POLD1 genes

Among 53 EC cases sequenced using Capture sequencing, no mutations were identified in the proofreading domains of *POLE* and *POLD1* genes. Among 379 EC cases analyzed by Sanger sequencing, four variants (1%, 4/379) were detected, two in *POLE* (0.53%) and two in *POLD1* (0.53%) proofreading domain and interpreted as of uncertain significance by ACMG/AMP 2015 guideline. Further analysis utilizing in silico pathogenicity prediction tools showed that all four were pathogenic mutations; c.1403A > G;p.468Y > C and c.940T > G;p.314S > A in *POLE* gene and c.1120G > A;p.374E > K and c.1231C > T;p.411Q > X in *POLD1* gene. Altogether, four variants (0.93%) were predicted to be pathogenic in 432 EC cases (Table 2).

**Table 2:** Characteristics of four mutations identified in our cohort

Gene	POLE	POLE	POLD1	POLD1
Mutation	c.940T > G:p.314S > A	c.1403A > G:p.468Y > C	c.1120G > A:p.374E > K	c.1231C > T:p.411Q > X
PolyPhen-2	Benign	Probably Damaging	Probably Damaging	NA
Mutation assessor	Medium	High	High	NA
SIFT	Tolerated	Damaging	Damaging	Damaging
CADD	22.1	27.1	33	37
Mutation Taster	Disease Causing	Disease Causing	Disease Causing	Disease Causing
Family history	NA	NA	Negative	Negative
Conservation between species	6 out of 6	6 out of 6	7 out of 7	3 out of 7
Age at diagnosis	62	69	55	75
Frequency in ExAC	0.00008245	0	0	0

The *POLE* p.314S > A is completely conserved and is also found in population database at a very low frequency of 0.00008 (ExAC). Another proofreading domain mutation p.468Y > C in *POLE* gene is also highly conserved and is completely absent in the population database of ExAC (Table 2).

The *POLD1* proofreading domain mutation, p.374E > K, was detected in a patient with early onset of EC. In addition, the p.411Q > X is partially conserved and p.374E > K is completely conserved in 6 species. Furthermore, these mutations are totally absent in the database of ExAC and were predicted as pathogenic by all five in silico prediction tools (Table 2).

Both cases harboring *POLE* mutations were older than 60 years, with one of them being serous (grade 3) and the other being endometrioid (grade 1) EC. The patient with *POLD1* p.374E > K mutation had grade 1 endometrioid EC. Another patient with *POLD1* p.411Q > X mutation was older than 60 years with grade 3 serous EC (Table 2). All the four cases harboring *POLE/POLD1* mutations were mismatch repair proficient as assessed by IHC.

### POLE and POLD1 expression in EC and their association with clinico-pathological features

**Fig. 1:**
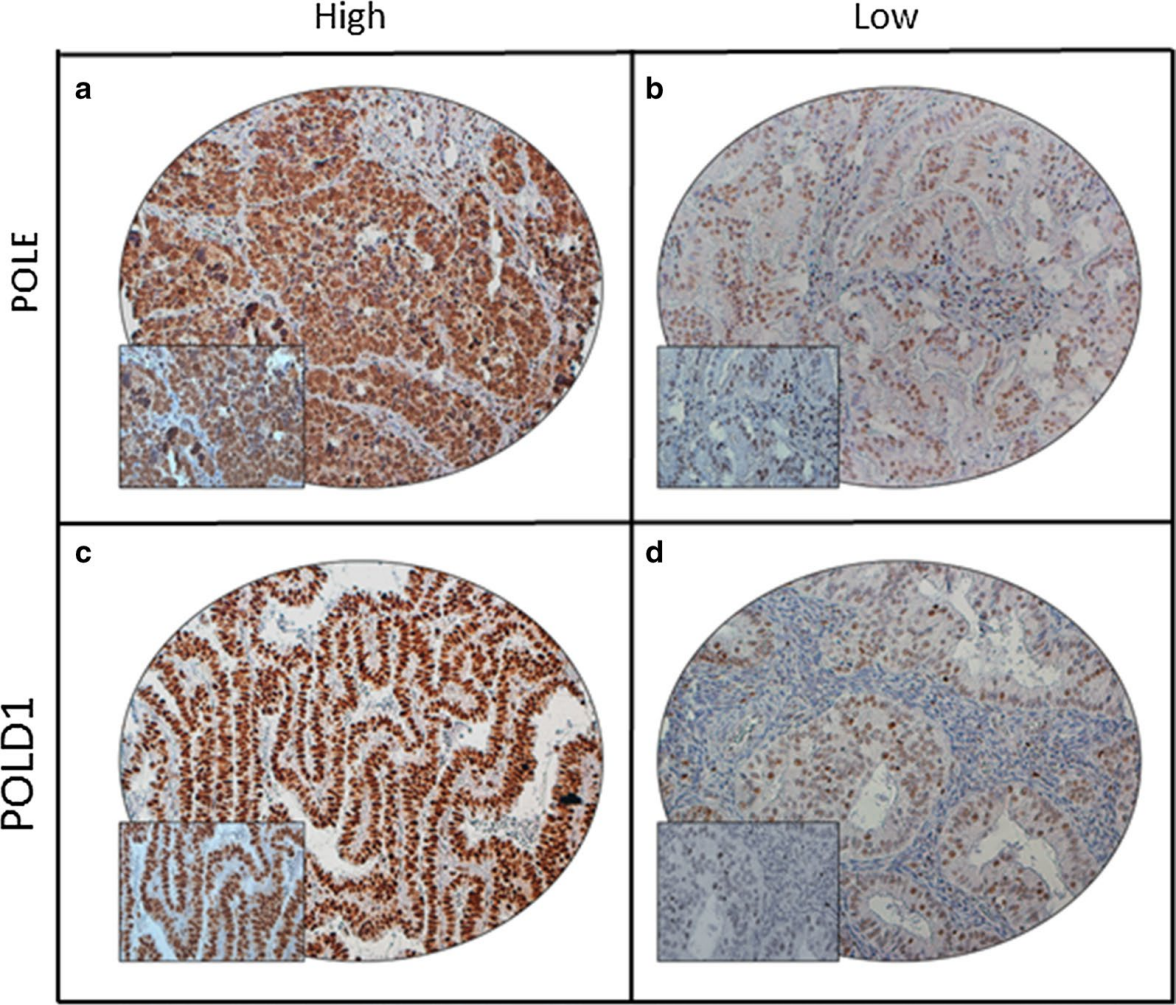
Tissue microarray based immunohistochemistry analysis of POLE and POLD1 in Endometrial carcinoma (EC) patients. EC TMA spots showing overexpression of POLE (**a**) and POLD1 (**c**). In contrast, another set of TMA spots showing reduced expression of POLE (**b**) and POLD1 (**d**). 20 X/0.70 objective on an Olympus BX 51 microscope (Olympus America Inc, Center Valley, PA, USA) with the inset showing a 40X 0.85 aperture magnified view of the same TMA spot

We next evaluated the expression of POLE and POLD1 by immunohistochemistry in 432 EC cases using tissue microarray. POLE immunohistochemical expression was interpretable in 414 cases. Low expression of POLE was noted in 41.1% (170/414) of cases and showed a significant association with grade 2 tumors (p = 0.0308). Both the cases harboring *POLE* mutation showed high nuclear expression of POLE protein. There was no significant association between POLE expression and microsatellite instability status (Table 3, Fig. 1a, b). POLD1 expression was interpretable in 419 cases. Low expression of POLD1 was noted in 59.9% (251/419) of cases and was significantly associated with grade 1 tumors (p = 0.0024) and a trend was noted with Type I EC (p = 0.0728). Of the two cases with *POLD1* mutation, one case showed high expression and another case showed low expression of POLD1 protein (Table 4, Fig. 1c, d).

**Table 3:** Association of clinico-pathological characteristics with POLE protein expression in Endometrial cancer

	Total		Low		High		p value
	No.	%	No.	%	No.	%	
No. of patients	414		170	41.1	244	58.9	
Age (years)							
≤ 50	76	18.4	33	43.4	43	56.6	0.6444
> 50	338	81.6	137	40.5	201	59.5	
Histologic subtype							
Type I	356	88.6	148	41.6	208	58.4	0.3741
Type II	46	11.4	16	34.8	30	65.2	
Lymphovascular invasion							
Present	88	27.4	39	44.3	49	55.7	0.2861
Absent	233	72.6	88	37.8	145	62.2	
Grade							
Grade 1	140	34.9	46	32.9	94	67.1	0.0308
Grade 2	138	34.4	66	47.8	72	52.2	
Grade 3	123	30.7	54	43.9	69	56.1	
pT							
T1	289	70.0	113	39.1	176	60.9	0.5879
T2	53	12.8	26	49.1	27	50.9	
T3	53	12.8	22	41.5	31	58.5	
T4	18	4.4	8	44.4	10	55.6	
pN							
N0	384	92.7	155	40.4	229	59.6	0.3051
N1–N2	30	7.3	15	50.0	15	50.0	
pM							
M0	392	94.9	163	41.6	229	58.4	0.2278
M1	21	5.1	6	28.6	15	71.4	
Stage							
I	267	64.7	104	39.0	163	61.0	0.5759
II	46	11.1	22	47.8	24	52.2	
III	66	16.0	30	45.5	36	54.5	
IV	34	8.2	13	38.2	21	61.8	
Microsatellite status							
MSI	52	12.6	25	48.1	27	51.9	0.2743
MSS	362	87.4	145	40.1	217	59.9	
POLE mutation							
Present	2	0.5	0	0.0	2	100.0	0.1452
Absent	412	99.5	170	41.3	242	58.7	
5 year overall survival				89.7		85.8	0.4691

**Table 4:** Association of clinico-pathological characteristics with POLD1 protein expression in Endometrial cancer

	Total		Low		High		p value
	No.	%	No.	%	No.	%	
No. of patients	419		251	59.9	168	40.1	
Age (years)							
≤ 50	78	18.6	47	60.3	31	39.7	0.9439
> 50	341	81.4	204	59.8	137	40.2	
Histologic subtype							
Type I	359	88.2	221	61.6	138	38.4	0.0728
Type II	48	11.8	23	47.9	25	52.1	
Lymphovascular invasion							
Present	90	27.7	51	56.7	39	43.3	0.4923
Absent	235	72.3	143	60.8	92	39.2	
Grade							
Grade 1	140	34.4	101	72.1	39	27.9	0.0024
Grade 2	140	34.4	77	55.0	63	45.0	
Grade 3	127	31.2	69	54.3	58	45.7	
pT							
T1	290	69.4	183	63.1	107	36.9	0.2590
T2	54	12.9	27	50.0	27	50.0	
T3	56	13.4	31	55.4	25	44.6	
T4	18	4.3	10	55.6	8	44.4	
pN							
N0	389	92.8	235	60.4	154	39.6	0.4491
N1–N2	30	7.2	16	53.3	14	46.7	
pM							
M0	396	94.7	240	60.6	156	39.4	0.3276
M1	22	5.3	11	50.0	11	50.0	
Stage							
I	268	64.1	171	63.8	97	36.2	0.1772
II	47	11.2	23	48.9	24	51.1	
III	68	16.3	38	55.9	30	44.1	
IV	35	8.4	19	54.3	16	45.7	
Microsatellite status							
MSI	52	12.4	34	65.4	18	34.6	0.3854
MSS	367	87.6	217	59.1	150	40.9	
POLD1 mutation							
Present	2	0.5	1	50.0	1	50.0	0.7767
Absent	417	99.5	250	59.9	167	40.1	
5 year overall survival				90.3		81.3	0.0555

## Discussion

Pathogenic mutations involving the proofreading domains of *POLE* and *POLD1* are widely known to be associated with colorectal polyposis and cancer [14, 36]. However, their role in EC is less well established. Here, we screened the proofreading domain of *POLE* and *POLD1* to detect causative variants in 432 unselected EC cases from the Middle Eastern region. We found two heterozygous mutations each in *POLE* (0.46%; 2/432) and *POLD1* genes (0.46%; 2/432). To the best of our knowledge, this is the first study to determine the frequency of germline *POLE* and *POLD1* mutations in EC from the Middle Eastern region. McConechy et al. [18] and Church et al. [19] have also reported a similar frequency for *POLE* and *POLD1* germline mutations in EC. A study from South East Asia reported a frequency of 4.3% each for *POLE* and *POLD1* germline mutations. However, the study was performed on only 47 selected cases of grade 3 endometrioid endometrial carcinomas [20] (Table 5). Other studies have reported the prevalence of POLE and POLD1 mutations at the somatic level, varying between 6.1 and 9.7% [12, 13, 37–39].

**Table 5:** Comparison of frequency of POLE and POLD1 mutations from different studies

Study	Year	Total cases	Frequency of germline POLE mutations (%)	Frequency of germline POLD1 mutations (%)
Our study	2019	432	0.46	0.46
Church et al. [19]	2013	173	0.58	0.58
Wong et al. [20]	2016	47	4.26	4.26
McConechy et al. [18]	2016	407	0.25	

Previous studies have shown that *POLE* proofreading-mutant cancers are a molecularly distinct group of tumors with a striking mutation burden and distinctive mutation signature [12, 19]. We have shown that *POLE* p.314S > A and p.468Y > C mutations are completely conserved between 6 species and found in population database at a very low frequency or absent respectively. Interestingly, this mutation (*POLE* p.314S > A) was predicted as colorectal carcinoma predisposing mutation in another study by our group (data unpublished). One of the *POLE* mutant cases was a grade 3 serous EC and the other was grade 1 endometrioid EC. Church et al. [19] also reported a single germline *POLE* mutation in grade 3 endometrioid EC. However, family history information of these mutation carriers are not available due to Middle Eastern conservative culture [40].

Two mutations in *POLD1*, p.374 E > K and p.411 Q > X, were also detected in patients with grade 1 endometrioid and grade 3 serous EC, respectively. These variants were not found in ExAC database. These mutations were partially conserved and predicted as pathogenic mutation by at least three in silico prediction tools. The *POLD1* p.411 Q > X mutation caused truncation of the protein in proofreading domain which would have adverse effect on the exonuclease activity of the gene, rendering this mutation highly pathogenic in nature. Consistent with previous reports, all the four cases harboring POLE or POLD1 mutations in our cohort were MSS tumors [12, 19, 41].

However, three out of four (75%) germline mutations identified were completely novel and weren’t reported previously in public database of ClinVar or other studies [42, 43], which could reflect the uniqueness of Saudi population (isolation, tribal origin and high consanguinity). The Gene Ontology (GO) analysis revealed POLE and POLD1 genes affect important biological processes including DNA replication proofreading and base-excision repair (Additional file 1: Table S1). It has been studied previously that loss of proofreading activity of replicative DNA polymerases and base-excision repair is responsible for various sporadic and hereditary cancers [44].

Several studies have reported favorable outcomes for women with *POLE*-mutated EC. This favorable prognosis has been attributed to the high number of mutations in tumors, expression of neoantigens, as well as an increase in patient immune responses [45]. Consistent with previous reports [18, 46], we observed no EC-related deaths or evidence of recurrent tumors in both patients with *POLE*-mutant cancers. However, we do acknowledge that the small number of tumors with *POLE* mutations limits our power, and therefore our results do not meet traditional levels of statistical significance. Despite this, our data contributes to the existing literature.

In this study, we showed that proofreading domain mutations in *POLE* and *POLD1* genes were not associated with protein expression of POLE and POLD1. This result could be partly explained by the fact that somatic proofreading domain mutations were not assessed. Interestingly, Campbell et al. [47] previously reported a low number of truncated mutations in proofreading domain as compared to the region outside of proofreading domain, and one-third of truncated *POLE* and *POLD1* mutations did not cause high tumor mutation burden. In addition, Elsayed et al. [48] also reported that the two *POLE* variant carriers in their cohort demonstrated positive POLE protein expression, which emphasizes the fact that POLE IHC does not have predictive value for effect of mutation. All these results indicated that POLE and POLD1 IHC analysis might not be suitable to select the patients for immunotherapy using immune checkpoint inhibitors.

## Conclusions

Our study shows a low frequency of germline mutations in *POLE* and *POLD1* proofreading domains in Middle Eastern EC patients. Although rare, screening for these mutations in individuals with high risk of developing EC might be clinically valuable. Since next generation sequencing technology offers significant benefits compared to single gene testing by reducing costs, time and increasing the sensitivity, it is feasible to screen multiple cancer related genes in EC patients using multigene panels including *POLE* and *POLD1*.

## Supplementary information

**Additional file 1: Table S1.** Functional analysis of POLE and POLD1 genes using Gene Ontology (GO).

## Data Availability

All data generated or analysed during this study are included in this published article.

## Abbreviations

EC: endometrial carcinoma
LS: lynch syndrome
POLE: polymerase epsilon
POLD1: polymerase delta 1
TCGA: The Cancer Genome Atlas
RAC: Research Advisory Council
FFPE: Formalin-Fixed Paraffin Embedded
PCR: polymerase chain reaction
TMA: tissue microarray
IHC: immunohistochemistry
